# Longitudinal *in vivo* biodistribution of nano and micro sized hydroxyapatite particles implanted in a bone defect

**DOI:** 10.3389/fbioe.2022.1076320

**Published:** 2022-12-19

**Authors:** Yang Liu, Sujeesh Sebastian, Jintian Huang, Tova Corbascio, Jacob Engellau, Lars Lidgren, Magnus Tägil, Deepak Bushan Raina

**Affiliations:** ^1^ Department of Clinical Sciences Lund, Orthopedics, The Faculty of Medicine, Lund University, Lund, Sweden; ^2^ Department of Hematology, Oncology and Radiation Physics, Skåne University Hospital, Lund, Sweden

**Keywords:** nano hydroxyapatite, micro hydroxyapatite, biodistribution, drug delivery, osteosarcoma

## Abstract

Hydroxyapatite (HA) has been widely used as a bone substitute and more recently as a carrier for local delivery of bone targeted drugs. Majority of the approved HA based biomaterials and drug carriers comprise of micrometer sized particulate HA (mHA) or granules and can therefore only be used for extracellular drug release. This shortcoming could be overcome with the use of cell penetrating HA nanoparticles (nHA) but a major concern with the clinical use of nHA is the lack of data on its *in vivo* biodistribution after implantation. In this study, we aimed to study the *in vivo* biodistribution of locally implanted nHA in a clinically relevant tibial void in rats and compare it with mHA or a combination of mHA and nHA. To enable *in vivo* tracking, HA particles were first labelled with ^14^C-zoledronic acid (^14^C-ZA), known to have a high binding affinity to HA. The labelled particles were then implanted in the animals and the radioactivity in the proximal tibia and vital organs was detected at various time points (Day 1, 7 and 28) post-implantation using scintillation counting. The local distribution of the particles in the bone was studied with micro-CT. We found that majority (>99.9%) of the implanted HA particles, irrespective of the size, stayed locally at the implantation site even after 28 days and the findings were confirmed using micro-CT. Less than 0.1% radioactivity was observed in the kidney and the spleen at later time points of day 7 and 28. No pathological changes in any of the vital organs could be observed histologically. This is the first longitudinal *in vivo* HA biodistribution study showing that the local implantation of nHA particles in bone is safe and that nHA could potentially be used for localized drug delivery.

## Introduction

Nanotechnology is an emerging cross-disciplinary scientific field that has been used to develop treatments for various diseases including cancer during the last decades (Ventola, 2012; Marques et al., 2019). Doxil^®^, the first FDA-approved nano-drug in 1995, was reported to improve anti-cancer efficacy with a better safety profile compared to conventional doxorubicin treatment (Barenholz, 2012; Zhao et al., 2018a). Likewise, Abraxane^®^, the nano-form of paclitaxel, was approved in 2005 and showed reduced side effects compared to the commonly used Taxol (Ma and Mumper, 2013). More recent advances in the field of nanomaterial-based drug delivery have been surface modification of lipid nanoparticles with ligand incorporation, thereby increasing tumor targeting without compromising the efficacy of loaded cytostatics (Tabernero et al., 2013; Kim et al., 2021). However, one of the prime drivers for better tumor targeting of nanomaterials containing cytostatics is the so-called enhanced permeability and retention (EPR) effect (Golombek et al., 2018). Existing studies indicate that only less than 5% of the systemically administered nanodrugs reach the target site *via* the circulation (Bae and Park, 2011; Wilhelm et al., 2016).

Bone is composed of 70% hydroxyapatite crystals and 30% organic components. The tightly organized structure formed by HA and collagen limits blood perfusion in bone when compared to other organs (Iranpour et al., 2016; Salles et al., 2021), which consequently leads to insufficient drug accumulation and efficacy especially in the cortical bone (Newman and Benoit, 2016). Previous studies indicated that systemically administered antibiotics such as ceftriaxone, flucloxacillin and gentamycin have poor penetration especially in the cortical compartment of long bones (Garazzino et al., 2011; Torkington et al., 2017). To overcome this shortcoming, carriers for local delivery of drugs targeting bone diseases have been widely explored for enhanced efficacy in fracture healing, deep infection and bone cancer (Stevens, 2008; Montoya et al., 2021; Sebastian et al., 2022). However, clinically available biomaterials used today for local drug delivery in bone are ceramic materials comprising of micrometer sized HA particles or poly methyl methacrylate (PMMA) cements (Pascual et al., 1996; Lawrie et al., 2020; Niemann et al., 2022). An important limitation with these materials is that they deliver the drug extracellularly. We recently demonstrated that the use of nano sized HA, loaded with a cornerstone osteosarcoma drug doxorubicin (DOX), can lead to intracellular cytostatic drug delivery and achieve significantly better tumor killing effect compared with systemically administered DOX. Others have shown that intracellular targeted drug delivery mediated *via* nanomaterials can enhance the treatment efficiency of bone diseases *via* the precise delivery of drugs to subcellular regions (Cheng et al., 2017).

Despite holding promise in targeted local delivery of cytostatics using nano carriers, the biological safety of using such nanomaterials would still pose a major concern (Zhao et al., 2018b). Drug loaded nanoparticles administered systemically may cause local inflammation and more importantly aggregate in vital organs causing damage (Ajdary et al., 2018; Najahi-Missaoui et al., 2020). Only a few studies, especially regarding bone, have explored the safety (local reaction and particle migration) aspects of using nHA alone or in combination of nHA and mHA (n/mHA) for local implantation (Kollenda et al., 2020). *In vivo* tracking of HA particles has been challenging because of the limitations associated with chemical coupling of molecular/imaging tags to the HA crystal.

We hypothesized that radioactively labelled zoledronic acid, a third generation bisphosphonate, could be used to form a rigid binding between ZA and HA, which would allow us to track the biodistribution of HA particles *in vivo*. The nHA, mHA and n/mHA particles were first reacted with ^14^C-ZA following which individual pellets of “labelled” particles were implanted in a metaphyseal bone void in the proximal tibia of rats to assess the radioactivity both in the bone and vital organs, which would indicate potential particle migration. To visualize the local HA migration within the bone, tibia implanted with HA particles were first subjected to micro-CT imaging followed by scintillation counting. To visualize the pathological changes in the vital organs that might be caused by nHA implantation and subsequent migration, an orthotopic xenograft osteosarcoma model was used to mimic the clinical scenario. Thus, the main aims of this study were to; 1. Explore the safety of using particulate nHA in the bone by tracking its *in vivo* migration over a period of 4-week and 2. The possibility of adding mHA with nHA to prevent particle migration.

## Methods

### Materials

Zoledronic acid (ZA) (Novartis, Switzerland) was purchased from the local pharmacy (Apoteket AB, Sweden) and was custom radiolabeled with ^14^C by a Perkin Elmer laboratory in United States. Hydroxyapatite (HA) powder was purchased from FLUIDINOVA, Portugal (micro hydroxyapatite powder, 10 μm and nano hydroxyapatite paste, <50 nm particle size), which were characterized in an earlier study (Liu et al., 2022) using XRD, SEM and TEM. The particles were sintered and crystalline and were in a size range of 30–50 nm in the case of nHA and 1–10 μm for mHA. The absorbable collagen membrane was a kind gift from Orthocell Ltd., Australia. Hematoxylin and Eosin (H&E) solutions were purchased from Thermo Scientific. Male Sprague-Dawley rats were purchased from Taconic (Denmark). 143B human osteosarcoma cells were purchased from American Type Culture Collection (ATCC). Athymic nude mice (Fox1^nu/nu^) were procured from Janvier Labs (France).

### Preparation of ^14^C-ZA labelled HA particles

The ^14^C-ZA solution was made by adding 1.62 ml ^14^C-ZA stock (1 mg/ml containing 7.1 MBq) to 13.5 ml PBS. In order to prepare labelled nHA particles, 750 mg nHA was mixed with 5 ml ^14^C-ZA solution for 24 h. Preparation of mHA and n/mHA mixture was made by weighing 562.5 mg mHA and a 50:50 wt% mixture of nHA (375 mg) and mHA (375 mg), respectively. Each particle type was then mixed with 5 ml ^14^C-ZA solution for 24 h. After reaction, the particles were centrifuged and supernatant was transferred to an Eppendorf tube. The particles were thoroughly washed twice with 5 ml PBS. The total disintegrations per minute (DPM) were measured in the original stock solution, the supernatant and the washed fractions using a scintillation counter (Wallac 1,414, PerkinElmer, United States), which was used to calculate the amount of ^14^C-ZA bound to HA.

### Materials characterization and HA pellet fabrication for *in vivo* implantation

The morphology of the HA particles was evaluated under a scanning electron microscope (SEM) (Jeol JSM-7800F, Sweden). The powder samples were dispersed on a SEM stub containing a double-sided carbon tape and imaged after sputter coating. The SEM instrument was operated at an operating voltage of 3.0 kV.

^14^C-ZA labelled HA particles (750 mg for nHA or n/mHA 246 and 562.5 mg for mHA) were mixed with approximately 300 µl hyaluronic acid (10 mg/ml) as a binder to create a HA paste, and the paste was casted into a sterile nylon mold (Ø = 3 mm) to produce pellets of defined dimensions. Each pellet contained 15 µl hyaluronic acid.

### Animal model

The bone defect in the proximal tibia was made according to our previously published protocol (Raina et al., 2019). All surgical procedures were conducted under isoflurane anesthesia and a pre-operative injection of buprenorphine (0.05 μg/kg). Animals also received antibiotic prophylaxis (intramuscular penicillin-streptocilin cocktail) before surgery. A metaphyseal bone defect was created in the proximal tibia by excising the skin below the medial side of the knee followed by dissecting the muscle tissue and scraping the periosteum to expose the proximal tibia. A hole (Ø = 3 mm) below the growth plate was made by a handheld drill and the hole was with covered with gauze until the wound stopped bleeding. Individual HA pellets (nHA, mHA or n/mHA) were then implanted in the defect in a press-fit manner. In order to prevent the local outward migration of the implanted particles into the soft tissue covering the hole, the bone defect containing the HA particles was covered with a collagen membrane (Ø = 4 mm). In total, 45 Sprague-Dawley (SD) male rats were operated which were divided into three groups: 1. nHA pellets (*n* = 15); 2. mHA pellets (*n* = 15) and 3. n/mHA pellets (*n* = 15). Five rats from each group were sacrificed and tissue samples (tibia, liver, spleen, kidney, heart, lung, brain and blood) collected at each time point (Day 1, 7 and 28 post-operation). One rat with mHA pellet died of anesthesia complications during the surgery.

### Micro-CT

Micro-CT analysis was performed on a MILabs U-CT system (Utrecht, Netherlands) to check the local distribution of the HA particles in the proximal tibia. The collected tibia specimens were transferred into a glass tube to prevent contamination, followed by placing the tibia parallel to the scanning bed. The imaging was performed with an ultra-focus scanning mode and a standard aluminum filter with the following x-ray tube settings: energy = 65 kV and current = 75 µA. After the scans were acquired, the raw images were reconstructed with an isotropic voxel size of 20 µm.

### Measurement of radioactivity in tissue specimens

Tissue specimens including the tibia, liver, spleen, kidney, heart, lung, brain and blood were collected from all rats sacrificed at day 1, 7 and 28 post-operatively. Total weight of each organ was measured on an analytical scale. The proximal tibia was cut using a Gigli wire after CT scan. All samples were placed in 5 ml Eppendorf tubes, filled with 2 ml PBS and homogenized using a sonication-based tissue homogenizer by introducing the probe into the tubes for approximately 60 s.

To be able to measure the radioactivity in bone, harvested and cut tibia were immersed in 3 ml 25% HCI solution to decalcify the bone tissue overnight. The samples were then homogenized as described above. 25 µl of the homogenized solution was taken and added to 2.5 ml scintillation liquid for further measurement. For other vital organs, 250 µl of the tissue homogenate from each tube was mixed with 2.5 ml scintillation liquid for detection of radioactivity. All scintillation vials were analyzed on a Wallac 1,414 scintillation counter (Perkin Elmer United States) for a scanning time of 60 s and the background readings of pure scintillation cocktail was subtracted from all specimens before calculating the radioactivity in each sample. Total DPM for each sample were normalized against the individual tissue weight and the radioactivity was expressed as DPM/g tissue. Three additional rats were operated without implanting HA particles or ^14^C-ZA administration, from which the proximal tibia and vital organs were taken as the “tissue specific” background controls. The DPM/g represents the radioactivity detected in the tested samples after weight normalization and subtraction of background counts from respective organs of normal animals (without the injection with ^14^C-ZA).

### H&E staining

Six athymic nude mice were first implanted with human osteosarcoma 143B cells in the proximal tibia to form an osteosarcoma. Ten days post cell inoculation, the matured tumor was debrided, which left a void in the bone. Six mice were divided into two groups: control group with no nHA particles (*n* = 3) and nHA group with nHA particles implanted in tibia defect after tumor debridement (*n* = 3). The liver, spleen, kidney, heart and lung were collected 21 days post-operation. The tissue was fixed with 4% formaldehyde for 24 h, followed by EDTA dehydration and paraffin embedding. Paraffin blocks were sectioned using a semi-automatic microtome (HM355S, Thermo Fisher Scientific, MA, United States) to a thickness of 5 μm and stained with H&E.

### Ethical permit

All animal procedures were approved (Ethical approval number 5.8.18–15288/2019 and 5.8.18–01018/2020) and performed in accordance with the directives of the Swedish regulatory authority for the use of animals for experimental purposes (Jordbruksverket).

### Statistic analysis

All data are presented as mean ± SD and checked for normality before statistical analysis. Kruskal–Wallis test with Dunn’s multiple comparisons was used to detect the differences in the DPM/g among various time points (D1, D7 and D28) in spleen and kidney. Paired *t*-test was used to detect the difference of DPM/g in the local proximal tibia site and the vital organs (Local vs. Systemic) for HA particles (nHA, mHA and n/mHA). All statistical analysis was carried out in Prism8 (GraphPad PRISM 8.2.1, United States) and *p* < 0.05 was considered statistically significant.

## Results

### The chemical binding of ^14^C-ZA to HA particles and its application within the study design

**FIGURE 1:**
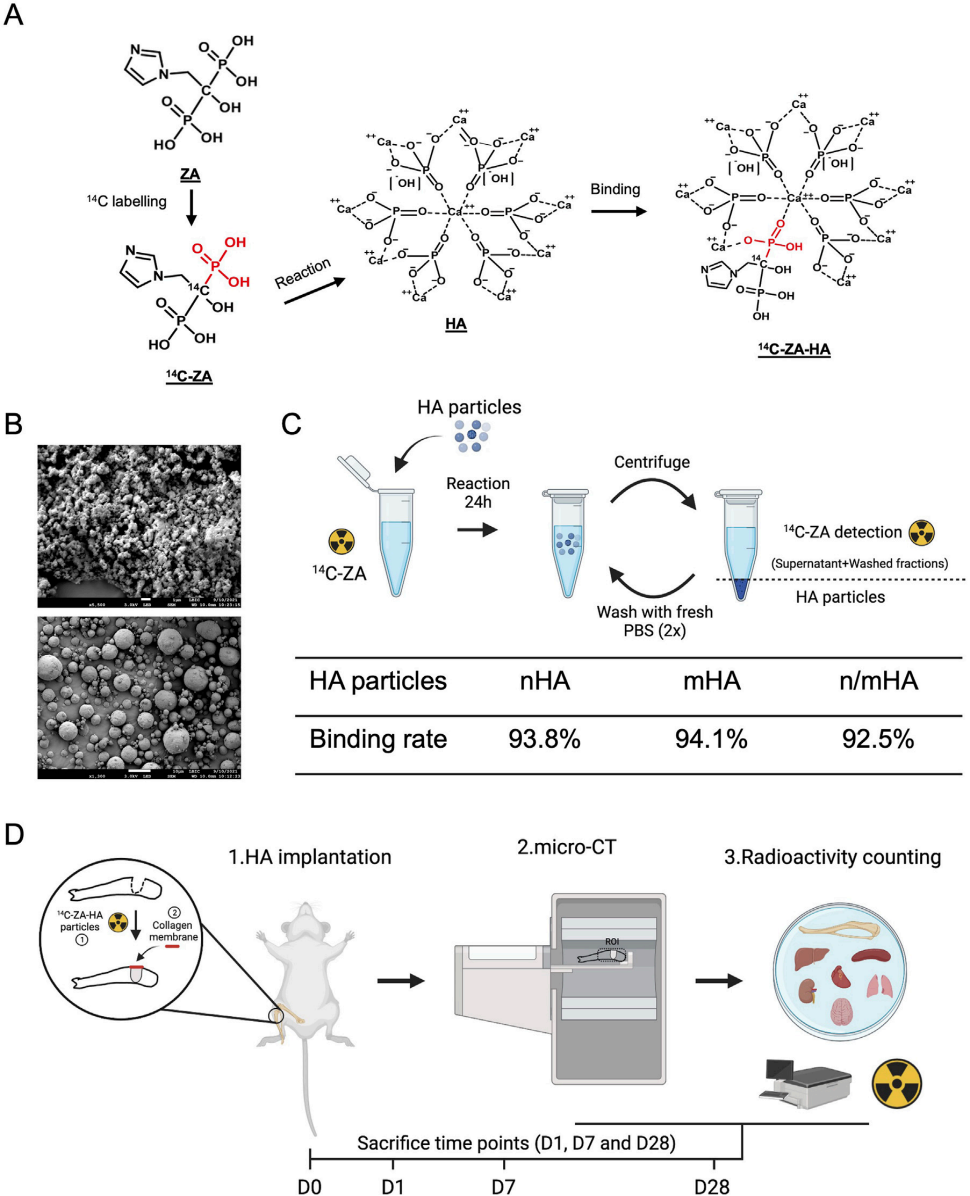
Hydroxyapatite particle characterization, labelling and the study design. **(A)** Shows the speculated mechanism of ^14^C-ZA and HA binding, which makes the evaluation of *in vivo* HA biodistribution possible. **(B)** Shows the shape and size distribution of nano- and micro-sized HA particles used in this study. **(C)** Schematic showing the process of ^14^C labelling for nHA, mHA and n/mHA and its binding rate. Panel C was made on BioRender.com. **(D)** Shows a schematic diagram of the timeline and the evaluation techniques used in the evaluation of *in vivo* HA particles biodistribution. Briefly, the HA particles (nHA, mHA and n/mHA) were fabricated into uniform pellets, which were implanted in the tibia defect and covered by a collagen membrane. At each time point (D1, D7 and D28), the tibia was scanned by micro-CT to detect the local distribution of implanted HA particles and collected samples (proximal tibia and vital organs) were further analyzed for the detection of radioactivity which indirectly indicated the biodistribution of ^14^C-ZA labelled HA particles. This figure was made on BioRender.com.

The speculated binding mechanism of ZA to HA particles has been described in Figure 1A and is based on earlier reports (Mukherjee et al., 2008), which indicate that the phosphate group in the HA crystal is substituted by the phosphate group on ZA, while the flanking hydroxyl group on ZA provides an electrostatic hook, which interacts with the HA calcium ions. The HA particles used in this study were characterized by SEM to verify their size distribution range. The nHA particles were rod/cube shaped with an average diameter of 50 nm and were seen as aggregates in the SEM images (Figure 1B, top). The mHA particles were arranged as uniform spheres with a diameter of approximately 1–10 μm (Figure 1B, bottom). The HA particles were weighed and reacted with ^14^C-ZA solution for 24 h as shown in the schematic in Figure 1C. After measuring the radioactivity in the stock solution, the supernatant and the washed fractions, the binding rate was calculated to be 93.8% for nHA, 94.1% for mHA and 92.5% for n/mHA, which indicated a strong chemical binding between ZA and HA (Figure 1C). The schematic of the study, timeline, and evaluation techniques are shown in Figure 1D.

### Fabrication of nHA, mHA and n/mHA pellets and *in vivo* implantation

**FIGURE 2:**
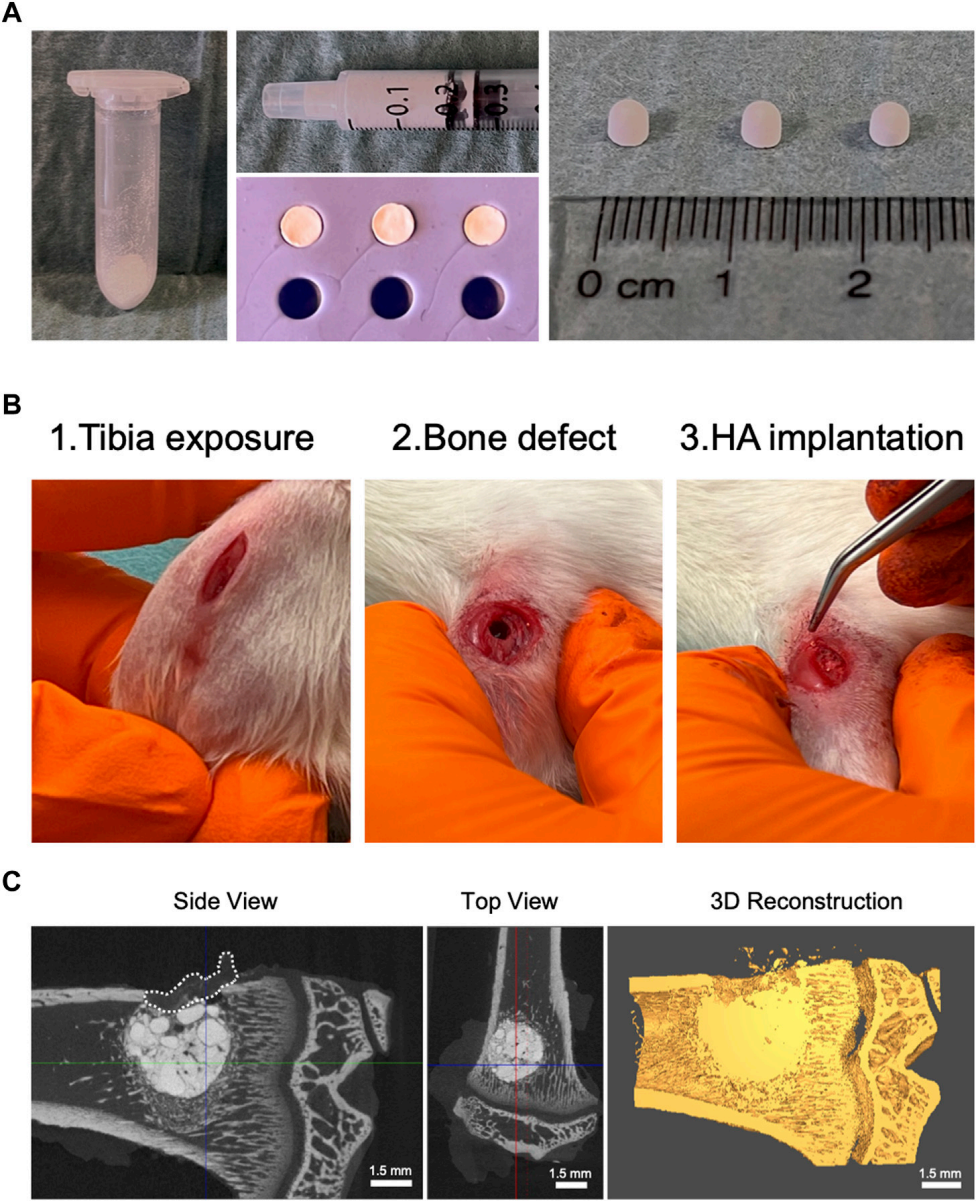
Labelled HA pellet fabrication and *in vivo* implantation. **(A)** Shows HA (nHA, mHA and n/mHA) particles first in the powder form (Left panel). After being mixed with hyaluronic acid, a HA paste was formed, which could be transferred into a syringe (Top panel, middle) and molded into pellets in a nylon mold (Top panel, middle). At the end, the fabricated pellets had a uniform hemispherical shape with a diameter of 3 mm (Right panel). **(B)** Shows the surgical procedure used during HA pellet implantation. **(C)** Shows the micro-CT images of the proximal tibia with the HA pellets implanted in a press-fit manner and the distribution of HA particles in the defect in a 3D reconstructed CT image. The white dashed line shows the placement of the collagen membrane.

To minimize HA particle loss during implantation and allow for the implantation of uniformly sized pellets to minimize variation, ^14^C-ZA labelled HA particles were mixed with 1% hyaluronic acid and casted into hemispherical pellets (Ø = 3 mm) (Figure 2A). After a period of 30 min, the pellets were recovered from the nylon mold and stored until *in vivo* implantation. Labelled HA pellets were implanted in a clinically relevant proximal tibial void covered with an absorbable collagen membrane (Figure 2B). Figure 2C shows a representative micro-CT image and its 3D reconstruction of a proximal tibia containing labelled HA pellet, which can be seen as press-fit into the bone void.

### The time kinetics of HA migration *in vivo*

**FIGURE 3:**
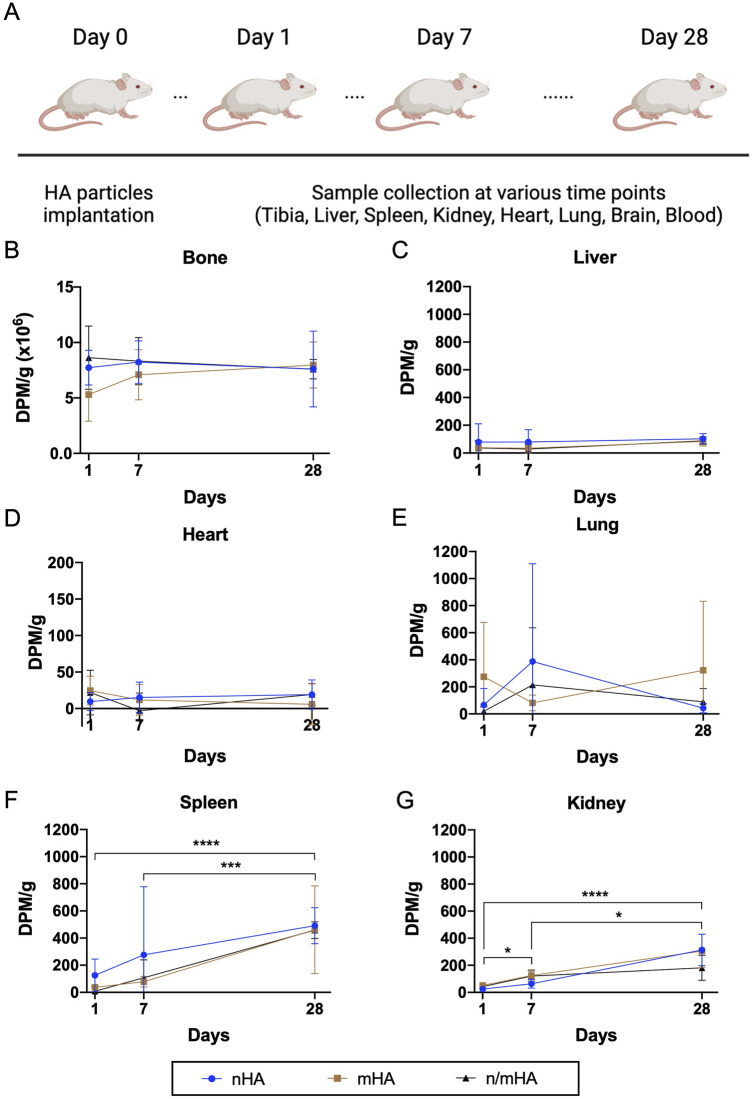
The time kinetics of HA particle (nHA, mHA and n/mHA) migration during the 28 days follow up period. **(A)** Shows follow-up timeline. **(B–G)** Shows the detected radioactivity of collected samples including the proximal tibia, liver, heart, lung, spleen and kidney, respectively. * indicates *p* < 0.05. *** indicates *p* < 0.001. **** indicates *p* < 0.0001.

Based on the long half-life of ^14^C, we followed the *in vivo* biodistribution of HA particles both locally as well as systemically in vital organs for up to 28 days (Figure 3A). Locally, within the proximal tibia where the HA particles were implanted, the level of radioactivity detected at day 1, 7 and 28 was high and did not differ significantly, irrespective of the pellet type (Figure 3B). Constant radioactive counts overtime indicated that the particles did not migrate from the implantation site. In the case of vital organs including liver, heart and lung, extremely low counts were detected and the counts were similar to the background values for the respective organs. Furthermore, no changes in the radioactive counts could be detected between day 1 and day 28 (Figures 3C–E). There was a minor increase in the radioactivity in spleen and kidney. The radioactivity in the spleen increased from 58.4 ± 98.9 (day 1) to 470.5 ± 190.6 (day 28) and in kidney from 38.8 ± 18.7 (day 1) to 267 ± 120.8 (day 28) (Figures 3F,G). This increasing trend of radioactivity could potentially indicate that some of the implanted particles or the free radioactive tag may have accumulated into the kidney and the spleen. However, when compared to the total radioactivity measured from particles implanted in the tibia, the signal detected in the kidney and spleen showed that <0.1% of implanted particles may have migrated to these organs. Finally, no radioactivity could be detected in the brain or the blood (Supplementary Figure S1).

### Differences in local and systemic migration of HA particles

**FIGURE 4:**
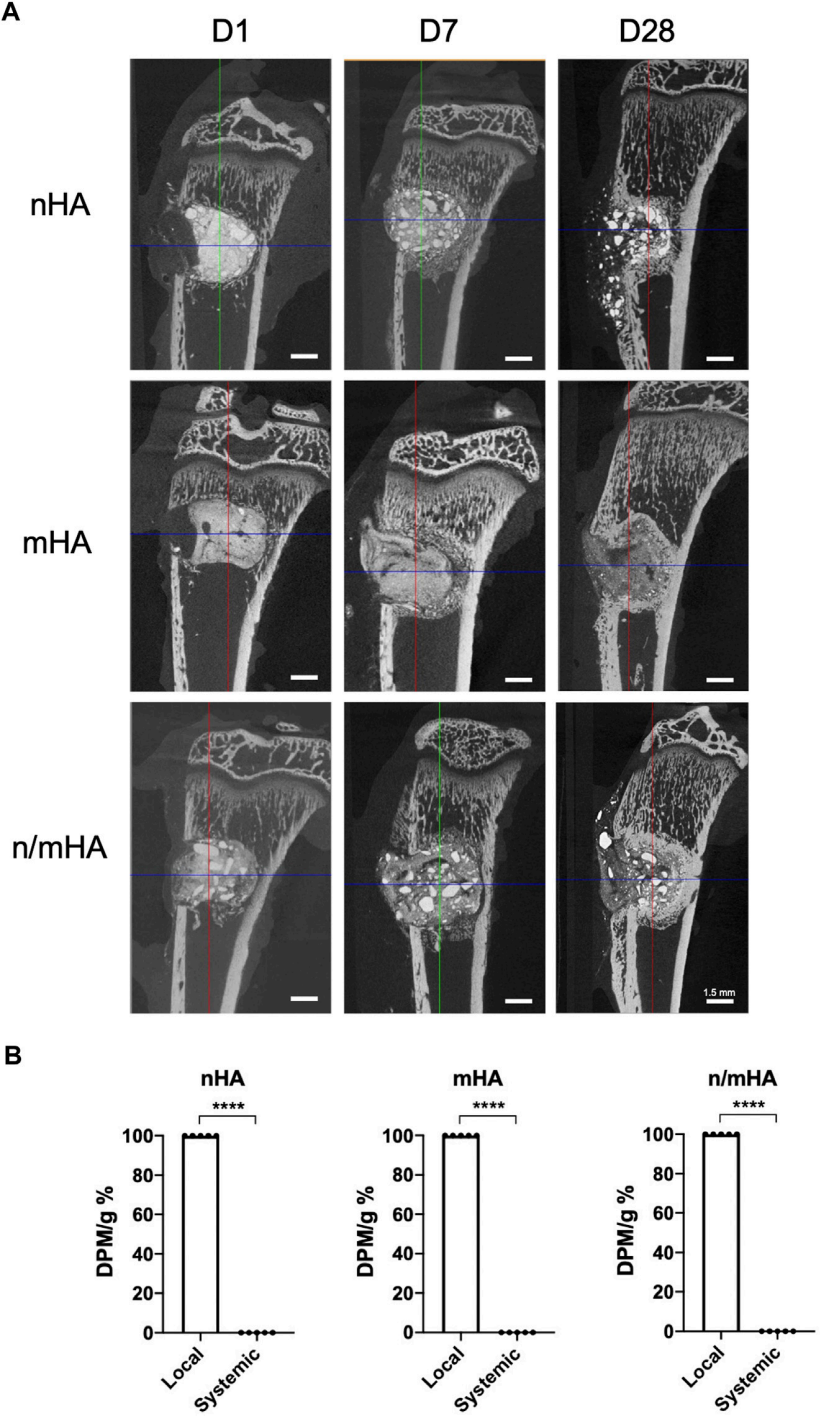
The local distribution of HA particles and its comparison to the amount of HA particles migrated systemically to vital organs. **(A)** Shows the micro-CT images of the local distribution of implanted HA particles in the proximal tibial over a course of 28 days **(B)** Shows the difference of detected radioactivity between the proximal tibia (local) and vital organs (systemic). **** indicates *p* < 0.0001.

To check the local distribution of HA particles, micro-CT was used to evaluate the visual differences in the proximal tibia after HA particle implantation over time. The implanted HA particles were mainly located inside the defect 1-day post-operation. There is a clear border between the implanted HA particles and the trabecular bone. After 7 days, some of the HA particles were pushed outside the cortex despite the presence of the collagen membrane. Around the implanted HA particles, tissues with trabecular-like structures could be observed with similar density to bone, which indicates the integration of HA particles within the bone defect. After 28 days, majority of the HA particles were retained inside the hole and potentially integrated with the new bone and some of the particles could be seen at the surface of the bone (Figure 4A). To compare the differences in radioactivity/particle migration from the local site (proximal tibia) to systemic distribution (vital organs), the percentage of the radioactivity counts in proximal tibia and vital organs were then calculated. It was observed that >99.9% of the radioactivity was retained within the proximal tibia and <0.1% radioactivity could be detected in other organs, irrespective of the particle size (Figure 4B).

### The biodistribution of HA particles in vital organs at early and late time point

**FIGURE 5:**
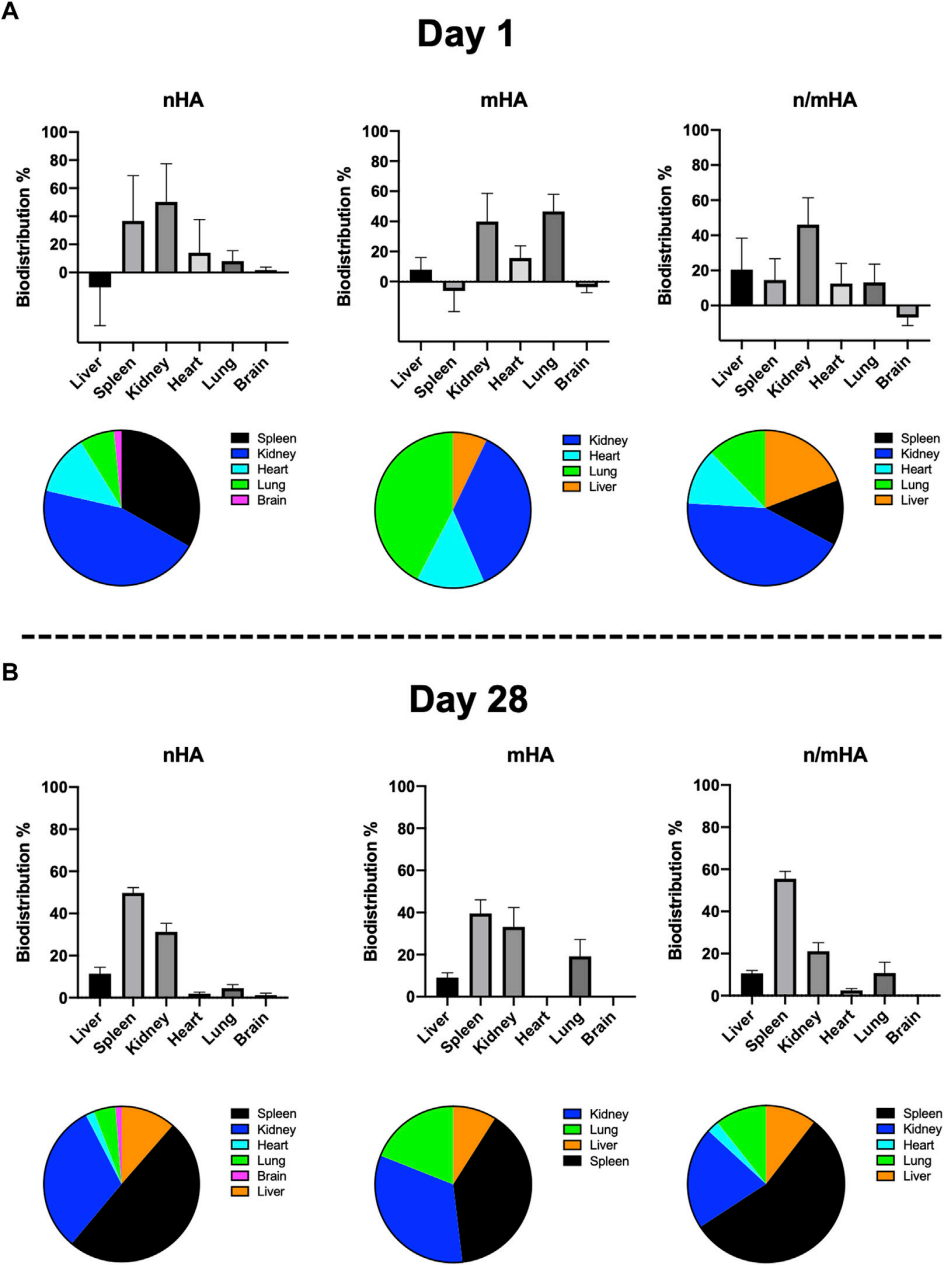
The proportion of HA particles which have migrated to vital organs at an early and late stage. **(A)** Shows the distribution of migrated HA particles in vital organs 1-day post-implantation. **(B)** Shows the distribution of migrated HA particles in vital organs 28-day post-implantation.

The relative particle biodistribution i. e radioactivity in vital organs including liver, spleen, kidney, heart, lung and brain were measured. At early time point (day 1), radioactivity was mainly detected in the spleen and kidney. For mHA, it was mainly in kidney and lung. However, when used as a combination, the majority of radioactivity was detected only in the kidney (Figure 5A). At the late time point (28 days), the majority of radioactivity were accumulated in spleen and kidney irrespective of the particle size (Figure 5B).

### Histological findings for vital organs

**FIGURE 6:**
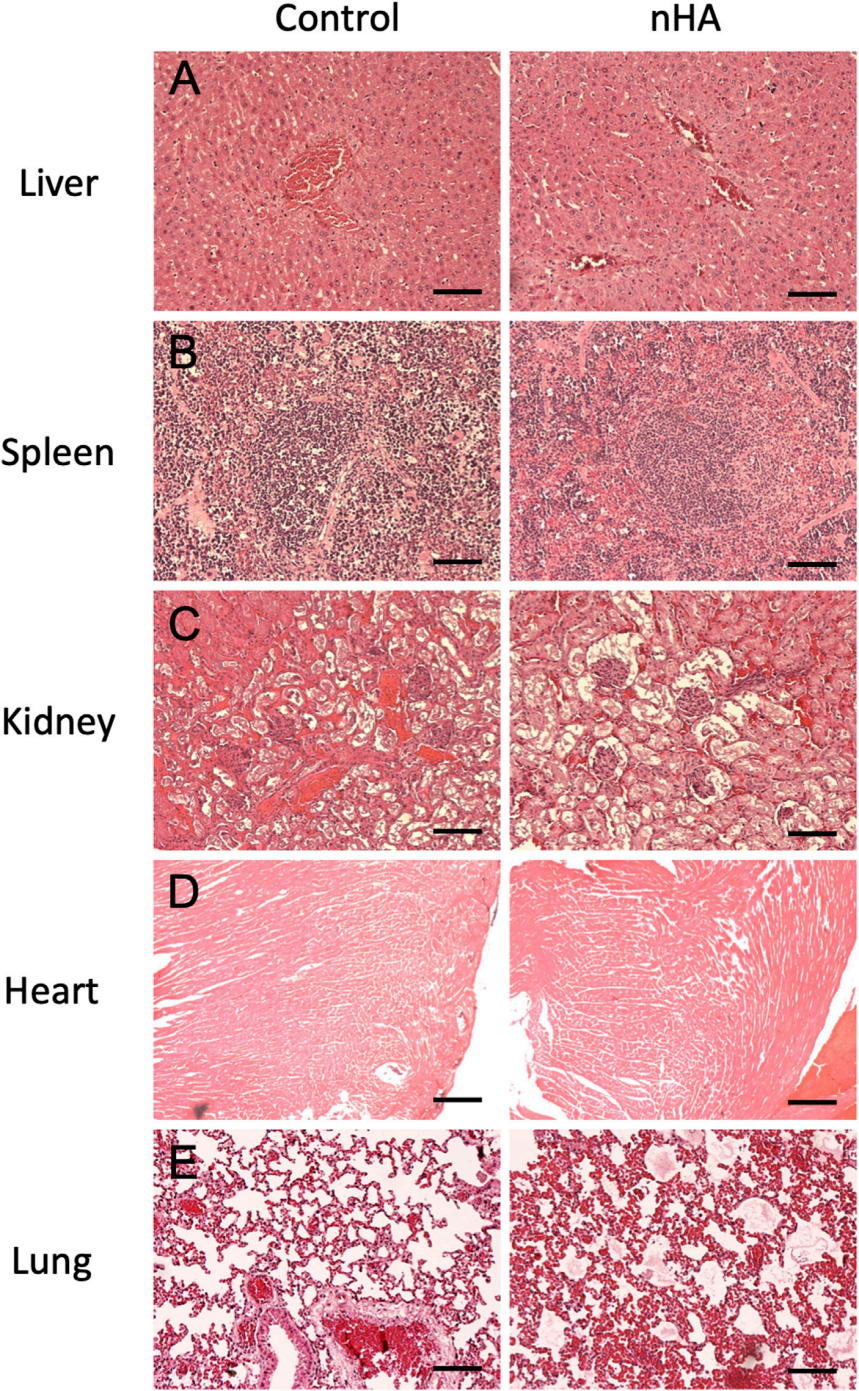
H and E staining of vital organs after HA particle implantation performed to detect the histopathological changes after local HA implantation. **(A–E)** Shows the microstructure of liver, spleen, kidney, heart and lung between control and nHA group. No differences could be observed in the two groups indicating that the local HA implantation did not cause histopathological changes in the vital organs in our model. Scale bar = 100 µm.

To further verify the pathological changes in vital organs and to detect the migrated HA particles, H&E staining was used to check the microstructure of the organs. In both control and nHA group, the hepatocytes were arranged in an organized pattern around the vein without any sign of apoptosis. There was no obvious difference in the neutrophil infiltration around the vein between the two groups, which indicated no inflammation caused by nHA (Figure 6A). In the spleen, the red and white pulp were clearly seen filled with immune cells in both groups (Figure 6B). The glomerulus and renal tubule were normal in sections from both groups, without any destruction in the microstructure of glomerulus (Figure 6C). Cardiac muscle fibers were oriented uniformly without any discontinuance. No obvious swelling or degeneration was seen in the muscle cells (Figure 6D). The typical porous microstructure was seen in the low magnification pictures in both groups. No immune cells were seen around the blood vessels in nHA group (Figure 6E).

## Discussion

Microparticulate HA based biomaterials have been approved and clinically used in patients with bone defects arising after fractures, infections and bone tumors (Fernandez de Grado et al., 2018; Horstmann et al., 2018). The systemic and local usage of HA nanoparticles has however been a subject of debate due to concerns regarding the risk of particle migration and accumulation leading to off-target cytotoxicity. In this study, we report that locally implanted nano, micro or a mixture of nano/micro-HA particles implanted in a bone void stay within the defect for up to 28 days after implantation.

HA is one of the most widely used biomaterial in the field of orthopedic surgery primarily due to its excellent biocompatibility and the ability to act as a template for bone regeneration. From a material production perspective, HA can be produced as granules or particles ranging from a few nanometers to several micrometers. nHA has demonstrated an improved osteoinductive effect on mesenchymal stem cells (MSC) compared to mHA (Wang et al., 2021). Furthermore, nHA has been elucidated to be cytotoxic to multiple cancer cells (Zhang et al., 2019; Liu et al., 2022). Owing to its chemical structure, HA has also been considered as an efficient carrier for drug delivery (Chen and Stephen Inbaraj, 2018). In an earlier study, we demonstrated that HA nanoparticles functionalized with DOX are first endocytosed and accumulated in endolysosomes, inducing a pH-dependent release of DOX. The intracellularly released DOX is then re-routed to the mitochondria instead of nucleus causing insufficient ATP synthesis, less cell migration and cell apoptosis (Liu et al., 2022). The superiority of delivering drugs by nHA over mHA has also been confirmed by *in vivo* studies (Salamanna et al., 2021; Liu et al., 2022), but potential and serious limitations of using HA nanoparticles are the risks of migration, accumulation and slow degradation in vital organs like liver, kidney or even brain (Hussain et al., 2013; Lee et al., 2013). For instance, gold nanoparticles have been shown to interact with Mac-1 and induce inflammation by activating NF-κB signaling pathway (Deng et al., 2011). The inflammation induced by gold nanoparticles was found in the lung with the presence of infiltrating lymphocytes and enhanced IL-1α expression in a rat study (Ng et al., 2016). Likewise, titanium dioxide (TiO_2_) nanoparticles, were found to be deposited in the heart causing myocardial injury (Sheng et al., 2013), in liver causing angiectasis and hyperaemia (Hong et al., 2014) and in spleen causing lymphocyte infiltration and fatty degeneration (Sang et al., 2013). Only one earlier study has explored the safety and biodistribution of nHA *in vivo* (Kollenda et al., 2020). Kollenda and co-workers modified calcium phosphate particles with 1,4,7,10-tetraazacyclododecane-1,4,7,10-tetrayl) tetraacetic acid (DOTA) to label calcium phosphate nanoparticles with ^68^Ga and track the *in vivo* biodistribution using SPECT-CT. They evaluated four different particle administration routes (intra-venous, intra-muscular, intra-tumoral and soft tissue) and reported that intravenous administration led to a rapid accumulation of nanoparticles in spleen, liver and lungs when compared to other particle administration routes. However, due to a short half-life of ^68^Ga (68 min), nanoparticle tracking was possible only during the first 4 hours of implantation, which could be considered rather short considering the long degradation time of HA based materials. To our knowledge, we present the first study of *in vivo* HA nanoparticle biodistribution with a 4-week follow-up.

*In vivo* tracking of HA particles has been challenging and in the authors opinion, caused by the difficulties in modifying the HA crystals, which make *in vivo* biodistribution studies challenging. To overcome this, we hypothesized that ^14^C-ZA could be used as a tracer to bind with HA particles. Zoledronic acid is a third generation bisphosphonate that is known to strongly bind HA *via* PO_4_^−^, Ca^2+^, and OH^−^ interactions, in which the PO_4_^3-^ on the HA crystal is replaced by the phosphate group (PO_3_) on the ZA molecule while the OH^−^ group on the ZA acts as an electrostatic hook with the calcium moiety on HA (Mukherjee et al., 2008)_._ In our previous studies, we have confirmed that ZA could also bind to synthetic HA, irrespective of the particle size. As a matter of fact, systemically administered ZA seeks and binds HA particles implanted in the muscle tissue of rats *in vivo* (Raina et al., 2020). It has been confirmed that the phosphate and hydroxyl groups in the bisphosphonates were responsible for ZA-HA binding, which could be modified to refine the binding affinity (Drake et al., 2008). In this study, we first labeled ZA with ^14^C, by replacing the central carbon atom with ^14^C without losing the bone seeking properties of the ZA molecule. This ^14^C-ZA was then reacted with HA particles for 24 h with a binding efficiency of >90%, which indeed confirmed that the radioactive labelling did not change the ZA-HA interaction. The long half-life of ^14^C (>5,000 years) allowed us to track the particles for a long period *in vivo* with high sensitivity. Other molecules have also been used to label ZA to detect drug accumulation (Verhulst et al., 2015; Swallow et al., 2018) but the possibility of using them as a tracer for HA particles needs to be explored further.

In order to mimic the clinical applications of HA particles being used as a void filler, we created a bone defect in the proximal tibia in rats using a well-established animal model (Raina et al., 2019). The proximal tibia in rats is highly vascularized and provides an environment that allows for systemic migration of implanted particles. Radioactively labelled HA particles were implanted into the defect of proximal tibia. The radioactivity of the collected tibia and vital organs was quantified as a surrogate marker for particle migration. We found that over 99% of implanted HA particles irrespective of size (nHA or mHA) stayed locally within the defect up to 28 days, indicating the safety of HA particle usage especially in the bone. Previous studies have shown inorganic nanoparticles could induce pathological changes in vital organs (Ferdous and Nemmar, 2020; Fadia et al., 2022). Nanoparticles that enter through the hepatic portal vein mostly reside in the liver due to the presence of leaky sinusoids and abundance of liver-resident cells with high endocytic or phagocytic activities (Tsoi et al., 2016). In spleen, it was reported that copolymer-coated nanoparticles (220 nm) could be internalized by the red-pulp and marginal zone macrophages (Moghimi et al., 1993). Various studies have shown nanoparticles with a diameter 20–100 nm tend to accumulate in the glomerulus when it reaches kidney (Dogra et al., 2018; Liu et al., 2020). For heart, nanoparticles have been found to accumulate in a long-term exposure, leading to sparse cardiac muscle fibers, inflammation, cellular necrosis, and cardiac biochemical dysfunction (Sheng et al., 2013). The deposition of nanoparticles in the lung can lead to chronic inflammation, epithelial injury, and further to pulmonary fibrosis (Byrne and Baugh, 2008). In our study, no pathological changes or inflammation was seen from the tissue samples from nHA group compared to control group.

One of the strategies to retain the nanoparticles locally is to embed them together with microparticles, which would stay at site for longer periods of time (Porsio et al., 2018). It has also been reported that the combination of nano- and micro-size HA shows less cytotoxic effects on healthy osteoblasts indicating better biocompatibility compared to only nanoparticles of HA (Liu et al., 2022). In this study, we implanted n/mHA composite in the tibial defect and from the biosafety profile of HA established in this study, biphasic HA could be a potentially efficient material for drug delivery in bone disorders. Owing to their size, nHA loaded with drugs such as antibiotics or cytostatics could be delivered intracellularly to specific cellular compartments of cancer cells or sessile bacteria hiding within the cells (Liu et al., 2022) (Qi et al., 2021). When used as a combination as nanoparticle-in-microparticle (NIM) system, multiple release profiles (burst release from outer particles and sustained release from internal components or intracellular release from nHA and extracellular release from mHA) can be envisaged. This phenomenon has been reported by Jelvehgari et al. who used theophylline loaded NIM to reduce the burst drug release of the outer microparticles (Jelvehgari et al., 2010). Apatite-binding drugs like tetracycline or doxorubicin could be loaded on NIM system for both intra- and extracellular drug delivery with better efficacy for combatting bone diseases such as tumors or infections (Wei et al., 2015; Raina et al., 2020; Lara-Ochoa et al., 2021). In addition, bone-targeting molecules could also be coupled with nanomaterials to increase their skeletal accumulation. Bioinspired exosomes or nanovesicles have been fabricated with abundant bone-targeting molecules present on their surface, showing 2–10 times more accumulation in the bone (mainly femur) compared to plain nanovesicles (Cui et al., 2022a; Cui et al., 2022b).

There are several limitations in this study. Firstly, the effect of osteoclasts on HA particles locally implanted within the bone has not been evaluated. The rheology for hyaluronic acid as a HA carrier and the effect on particle migration have not been evaluated. However, the small quantity of hyaluronic acid used/pellet as well as its rapid clearance from the body makes it a tentative carrier candidate for HA based particulate materials. Secondly, despite a high and strong binding of ZA to HA, the amount of chemically bound ZA released from HA particles into the systemic circulation, which is either mediated by osteoclasts or acidic environment could not be differentiated from actual nanoparticle migration. However, due to extremely low radioactivity detected in other vital organs, it is feasible to conclude that neither the labelled nanoparticles nor the free ZA was detected systemically. Lastly, biochemical biomarkers of vital organ functionality/damage due to nanoparticle migration that could assist in further strengthening the conclusions regarding the biosafety of HA particles were not studied.

## Conclusion

By labelling HA with ^14^C-ZA, nHA alone or in combination with mHA showed a promising safety profile during the course of this *in vivo* biodistribution study, thereby strengthening the case for the local use of nHA in bone defects. The HA nano-in-micro platform as drug delivery carrier should be explored further allowing clinical translation using apatite-binding drugs in the treatment of bone tumors and osteomyelitis.

## Data Availability

The raw data supporting the conclusions of this article will be made available by the authors, without undue reservation.
