# Supplementary material for: Longitudinal in vivo biodistribution of nano and micro sized hydroxyapatite particles implanted in a bone defect
Source: Front Bioeng Biotechnol. 2022 Dec 19;10:1076320. doi: 10.3389/fbioe.2022.1076320 (PMC9806272; doi:10.3389/fbioe.2022.1076320)
Supplement: Supplementary file 1 [file DataSheet1.doc]

Supplementary Materials for

**Longitudinal in-vivo biodistribution of nano and micro sized hydroxyapatite particles implanted in a bone defect**

Yang Liu, Sujeesh Sebastian, Jintian Huang, Tova Corbascio, Jacob Engellau, Lars Lidgren, Magnus Tägil, Deepak Bushan Raina

*Corresponding author. Email: [liu.yang@med.lu.se](mailto:liu.yang@med.lu.se), deepak.raina@med.lu.se

**This PDF file includes:**

Figs. S1


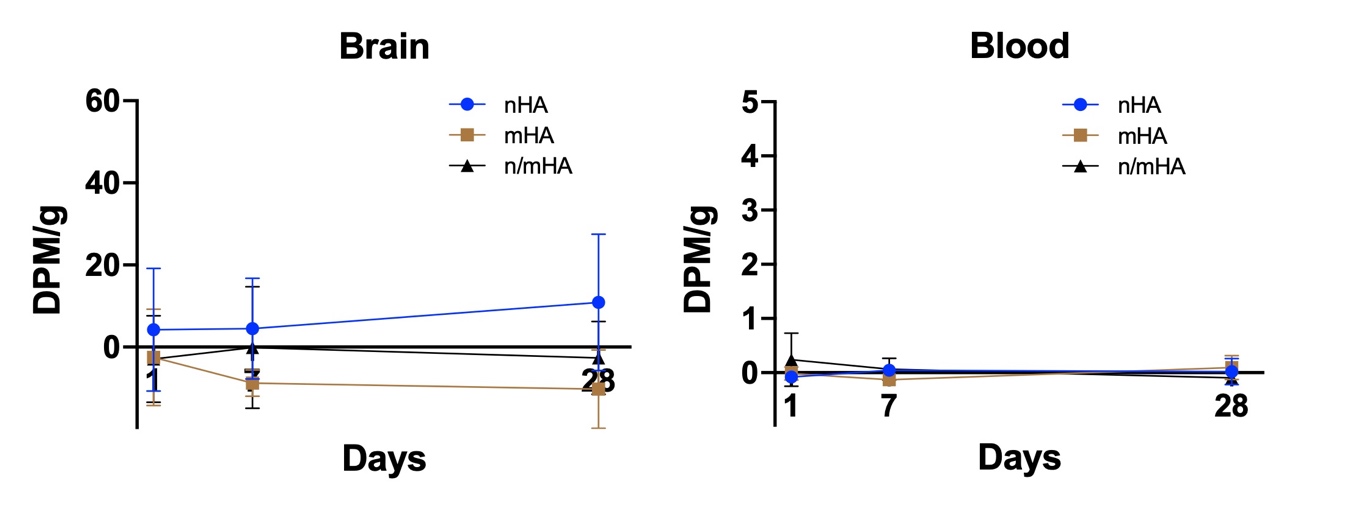


Supplementary Figure S1. The time kinetics of HA particle (nHA, mHA and n/mHA) migration in the brain and blood during the 28 day follow up period.
